# A Survey of Wireless Sensor Network Based Air Pollution Monitoring Systems

**DOI:** 10.3390/s151229859

**Published:** 2015-12-12

**Authors:** Wei Ying Yi, Kin Ming Lo, Terrence Mak, Kwong Sak Leung, Yee Leung, Mei Ling Meng

**Affiliations:** 1 Department of Computer Science and Engineering, The Chinese University of Hong Kong, Shatin NT, Hong Kong, China; wyyi1991@gmail.com (W.Y.Y.); kmlo@cse.cuhk.edu.hk (K.M.L.); 2 Institute of Future Cities, The Chinese University of Hong Kong, Shatin NT, Hong Kong, China; yeeleung@cuhk.edu.hk; 3 Department of Electronics and Computer Science, University of Southampton, University Road, Southampton S017 1BJ, UK; tmak@ecs.soton.ac.uk; 4 Department of Geography and Resource Management, The Chinese University of Hong Kong, Shatin NT, Hong Kong, China; 5 Department of Systems Engineering and Engineering Management, The Chinese University of Hong Kong, Shatin NT, Hong Kong, China; hmmeng@se.cuhk.edu.hk; 6 Stanley Ho Big Data Decision Analytics Research Center, The Chinese University of Hong Kong, Shatin NT, Hong Kong, China

**Keywords:** air pollution monitoring, Wireless Sensor Network (WSN), real-time monitoring, high spatio-temporal resolution, low-cost ambient sensor

## Abstract

The air quality in urban areas is a major concern in modern cities due to significant impacts of air pollution on public health, global environment, and worldwide economy. Recent studies reveal the importance of micro-level pollution information, including human personal exposure and acute exposure to air pollutants. A real-time system with high spatio-temporal resolution is essential because of the limited data availability and non-scalability of conventional air pollution monitoring systems. Currently, researchers focus on the concept of The Next Generation Air Pollution Monitoring System (TNGAPMS) and have achieved significant breakthroughs by utilizing the advance sensing technologies, MicroElectroMechanical Systems (MEMS) and Wireless Sensor Network (WSN). However, there exist potential problems of these newly proposed systems, namely the lack of 3D data acquisition ability and the flexibility of the sensor network. In this paper, we classify the existing works into three categories as Static Sensor Network (SSN), Community Sensor Network (CSN) and Vehicle Sensor Network (VSN) based on the carriers of the sensors. Comprehensive reviews and comparisons among these three types of sensor networks were also performed. Last but not least, we discuss the limitations of the existing works and conclude the objectives that we want to achieve in future systems.

## 1. Introduction

Over the past few years, air pollution has drawn a lot of interest in terms of research and everyday life. According to data from Google Search, about 46 million results are related to “2014 Air Pollution”, while the number of results related to “2014 Nobel Prize” is only about 27 million (accessed on 2014-8-20). The public concern on air pollution increases significantly due to the serious hazards to the public health, as described in [1]. Heart disease, Chronic Obstructive Pulmonary Disease (COPD), stroke and lung cancer are highly related to air pollution. People breathing in air of poor quality could suffer from difficulty in breathing, coughing, wheezing and asthma. In addition to the human health, air pollution also has a major effect on the global environment and the worldwide economy. It is well known that acid rain, haze and global climate change are caused by air pollution. In 2010, the European Commission threatened the UK with legal actions for the breaching of PM_10_ (PM_X_ stands for particulate matter with diameter of less than or equal to X μm) limit values. The UK could pay £300 million per year for this [2].

In order to mitigate the impacts of air pollution on human health, global environment and worldwide economy, governments have put tremendous efforts on air pollution monitoring. With detailed information of the air pollution situation, scientists, policy makers and planners are able to make informed decisions on managing and improving the living environment [3]. Countries adopting proper policies on air pollution can reduce the public health expenses as described above [4].

Traditionally, air pollution situation is monitored by conventional air pollution monitoring systems with stationary monitors. These monitoring stations are highly reliable, accurate and able to measure a wide range of pollutants by using the conventional analytical instruments, such as gas chromatograph-mass spectrometers [5].

**Figure 1:**
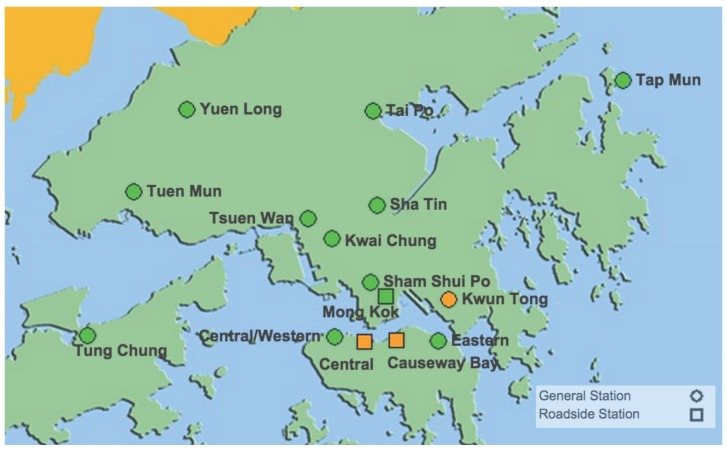
Deployment of stationary monitors in Hong Kong [6].

**Table 1:** The number of stationary monitors in selected cities.

City	Number of Stationary Monitors	Coverage Area	Coverage Per Monitor (Number of Football Fields)
Beijing, China	35 [7]	16,000 km^2^	64,025
Hong Kong, China	15 [6]	2700 km^2^	25,210
New York, USA	44 [8]	1200 km^2^	3820
London, UK	123 [9]	1600 km^2^	1822

The drawbacks of the conventional monitoring instruments are their large size, heavy weight and extraordinary expensiveness. These lead to sparse deployment of the monitoring stations (see Figure 1 and Table 1). In order to be effective, the locations of the monitoring stations need careful placement because the air pollution situation in urban areas is highly related to human activities (e.g., construction activities) and location-dependent (e.g., the traffic choke-points have much worse air quality than average) [10,11,12]. Changes in urban arrangement, activities or regulation may affect both the species and the concentrations of air pollutants, which require relocating stations or adding new stations. These requirements are typically hard or even impossible to fulfill due to the cost inefficiency in acquisition and maintenance of the monitoring stations. Moreover, the conventional monitoring instruments involve long-term time-consuming average models. The air pollution situation is updated hourly or even daily. Hence, the air pollution maps built by the conventional air pollution monitoring systems are with extremely low spatial and temporal resolutions.

Such low spatio-temporal resolution is sufficient for ambient background monitoring but extremely inadequate for the public to be aware of their personal exposure to air pollution and cannot reflect their personal health risks. In [13], researchers noted that the pollutant concentrations within a street may vary over a space with magnitude of few meters and over time with magnitude of few seconds. The conventional monitoring systems cannot detect this phenomenon because of their limited data availability and non-scalability characteristics. Furthermore, when road traffic is the major pollution source, which is always the case in urban areas, acute exposure to the public is prevalent [14]. Evidences show that acute exposure to or short-term change of pollutants may trigger or worsen some health events or diseases [15,16,17].

In order to increase the spatio-temporal resolution of the air pollution information, researchers are pushing the air pollution monitoring systems to the limit by combining the low-cost portable ambient sensors and the Wireless Sensor Network (WSN) into one system which is known as The Next Generation Air Pollution Monitoring System (TNGAPMS) [18]. By utilizing the low-cost portable ambient sensors and the WSN, the air pollution information can be updated in minutes or even seconds [19]. Also, the low-cost portable sensors enable the mobility and the feasibility in large-scale deployment of the sensor nodes. The spatial and temporal resolutions of the pollution information are significantly increased in TNGAPMS. TNGAPMS fills the gap between the conventional monitoring systems and the air quality models because the air pollution information at locations without monitoring stations is accomplished by air quality models or estimations [20]. TNGAPMS also helps researchers understand the distribution of the air pollutants more efficiently and accurately to improve the air quality models. The public users can even measure their personal exposures to pollutants using wearable sensor nodes [21].

Researchers anticipate that the real-time, high spatio-temporal (The spatial resolution of the air pollution information is in scale of tens to hundreds of square meters while the air pollution information of a specific location has reporting interval less than few minutes and is available to the users, including researchers, public users, and policy makers, with minimal or no delay.) air pollution information can help advise the public to take proper actions according to their individual health needs (e.g., asthmatics could choose an alternative healthier route to minimize the personal air pollution exposure), and raise public awareness about the air pollution that further leads to change of public “unclean” activities (e.g., driver with better driving habits can reduce pollutants’ emission).

The remainder of this paper is organized as follows. In Section 2, the air quality standards defined by different agencies all over the world are introduced. In Section 3, we discuss the limitations of the measurement equipment used in conventional air pollution monitoring systems and the opportunities provided by the low-cost portable ambient sensors. In Section 4, 20 state-of-the-art systems of TNGAPMS are presented and classified into three categories. The advantages and disadvantages of each category are described. In Section 5, we focus on the comparisons of the three categories of existing works classified in Section 4. Finally, we point out the limitations of the existing works and conclude the objectives we want to achieve when building a future TNGAPMS in Section 6.

## 2. Air Quality Standards

Pollutants are emitted by human activities and natural sources. Hundreds of hazardous pollutants in our living environment have been identified [22]. However, six of these pollutants are well studied and ubiquitous in our daily lives, including carbon monoxide (CO), nitrogen dioxide (NO_2_), ground level ozone (O_3_), sulfur dioxide (SO_2_), particulate matter (PM) and lead (Pb) [23]. The health effects (see Table 2) and environmental effects caused by these pollutants can be found in [24,25,26,27,28,29].

**Table 2:** The six common pollutants and their health effects.

Pollutant	Health Effects
Carbon Monoxide (CO)	Reducing oxygen capacity of the blood cells leadsto reducing oxygen delivery to the body’s organsand tissues. Extremely high level can cause death.
Nitrogen Dioxide (NO_2_)	High risk factor of emphysema, asthma andbronchitis diseases. Aggravate existing heartdisease and increase premature death.
Ozone (O_3_)	Trigger chest pain, coughing, throat irritationand congestion. Worsen bronchitis, emphysemaand asthma.
Sulfur Dioxide (SO_2_)	High risk factor of bronchoconstrictionand increase asthma symptoms.
Particulate Matter (PM_2.5_ & PM_10_)	Cause premature death in people with heart andlung diseases. Aggravate asthma, decrease lungfunction and increase respiratory symptomslike coughing and difficulty breathing.
Lead (Pb)	Accumulate in bones and affect nervous system,kidney function, immune system, reproductivesystems, developmental systems and cardiovascularsystem. Affect oxygen capacity of blood cells.

Governments and organizations have put regulation limits on these pollutants to reduce the risks. The United States Environmental Protection Agency (EPA), the World Health Organization (WHO), the European Commission (EC), the Chinese Ministry of Environmental Protection (MEP) and the Environmental Protecting Department (EPD) of Hong Kong have declared different standard limits for these pollutants (see Table 3).

In order to help the public understand the current air quality easily, the government and organization agencies introduced an indicator called Air Quality Index (AQI). AQI measures the “condition or state of each relative to the requirements of one or more biotic species and/or to any human need or purpose” [30]. In a word, it tells the public how “good” the current air quality is or the forecast air quality will be. Different agencies may use different air quality indices [31,32,33,34].

To illustrate the concept of AQI, an AQI example introduced by the Environmental Protection Department (EPD) of Hong Kong [35] called Air Quality Health Index (AQHI) system is given (see Table 4). The AQHI system provides a better understanding on health risks to the public and suggests detail precautionary actions with respect to each AHQI level [36].

**Table 3:** Different standards of the six common pollutants.

Pollutant		EPA [37]	WHO [38,39,40]	EC [41]	MEP [42]	EPD [43]
Carbon Monoxide (CO)		9 ppm (8 h) 35 ppm (1 h)	100 mg/m^3^ (15 min) 15 mg/m^3^ (1 h) 10 mg/m^3^ (8 h) 7 mg/m^3^ (24 h)	10 mg/m^3^ (8 h)	10 mg/m^3^ (1 h) 4 mg/m^3^ (24 h)	30 mg/m^3^ (1 h) 10 mg/m^3^ (8 h)
Nitrogen Dioxide (NO_2_)		100 ppb (1 h) 53 ppb (1 year)	200 μg/m^3^ (1 h) 40 μg/m^3^ (1 year)	200 μg/m^3^ (1 h) 40 μg/m^3^ (1 year)	200 μg/m^3^ (1 h) 80 μg/m^3^ (24 h) 40 μg/m^3^ (1 year)	200 μg/m^3^ (1 h) 40 μg/m^3^ (1 year)
Ozone (O_3_)		75 ppb (8 h)	100 μg/m^3^ (8 h)	120 μg/m^3^ (8 h)	200 μg/m^3^ (1 h) 160 μg/m^3^ (8 h)	160 μg/m^3^ (8 h)
Sulfur Dioxide (SO_2_)		75 ppb (1 h) 0.5 ppm (3 h)	500 μg/m^3^ (10 min) 20 μg/m^3^ (24 h)	350 μg/m^3^ (1 h) 125 μg/m^3^ (24 h)	500 μ g/m^3^ (1 h) 150 μg/m^3^ (24 h) 60 μg/m^3^ (1 year)	500 μg/m^3^ (10 min) 125 μg/m^3^ (24 h)
Particulate Matter	PM_2.5_	35 μg/m^3^ (24 h) 12 μg/m^3^ (1 year)	25 μg/m^3^ (24 h) 10 μg/m^3^ (1 year)	25 μg/m^3^ (1 year)	75 μg/m^3^ (24 h) 35 μg/m^3^ (1 year)	75 μg/m^3^ (24 h) 35 μg/m^3^ (1 year)
	PM_10_	150 μg/m^3^ (24 h)	50 μg/m^3^ (24 h) 20 μg/m^3^ (1 year)	50 μg/m^3^ (24 h) 40 μg/m^3^ (1 year)	150 μg/m^3^ (24 h) 70 μg/m^3^ (1 year)	100 μg/m^3^ (24 h) 50 μg/m^3^ (1 year)
Lead (Pb)		0.15 μg/m^3^ (3 month)	0.5 μg/m^3^ (1 year)	0.5 μg/m^3^ (1 year)	1 μg/m^3^ (3 month) 0.5 μg/m^3^ (1 year)	1 μg/m^3^ (3 month) 0.5 μg/m^3^ (1 year)

**Table 4:** Air Quality Health Index (AQHI) of Hong Kong Environmental Protection Department.

Health Risk Category	AQHI
Low (Green)	1
	2
	3
Moderate (Orange)	4
	5
	6
High (Red)	7
Very High (Brown)	8
	9
	10
Serious (Black)	10+

## 3. Air Pollution Monitoring Equipment

Conventional air pollution monitoring systems are mainly based on sophisticated and well-established instruments. In order to guarantee the data accuracy and quality, these instruments use complex measurement methods [44] and a lot of assisting tools including temperature controller (cooler and heater), relative humidity controller, air filter (for PM), and build-in calibrator [45]. As consequences, these instruments are typically with high cost, high power consumption, large volume, and heavy weight. Thanks to technology advance, ambient sensors with low cost, small size and fast response time (in the order of seconds or minutes) is available recently. However, no low-cost portable ambient sensor can achieve the same data accuracy and quality as conventional monitoring instruments [46] (see Table 5 and Table 6).

Currently, the air pollution data at locations without monitoring stations are obtained by air quality models or estimations [20]. However, the data from the air quality models lack of cross-validation and verification. The low-cost portable ambient sensors provide a huge opportunity in increasing the spatio-temporal resolution of the air pollution information and are even able to verify, fine-tune or improve the existing ambient air quality models.

In the following subsections, the working mechanisms of the low-cost portable ambient sensors that are widely used in TNGAPMS are introduced. As a matter of fact, except the air pollution detecting technologies mentioned in Section 3.1 and Section 3.2, there are other detecting technologies such as Surface Acoustic Wave (SAW) [47,48,49], Quartz Tuning Fork (QTF) [50,51], Raman Lidar [52,53] and Differential Ultra Violet Absorption Spectroscopy (DUVAS) [54,55] that we will not discuss for unpopularity reason.

**Table 5:** Instruments used in air quality monitoring systems (Part A).

Pollutant		Example Product	Measurement Method	Resolution	Accuracy	Range	Price (USD)
PM_2.5_		Met One Instrument BAM-1020 Beta Attenuation Monitor [56]	Beta Attenuation	1 μg/m^3^	±1 μg/m^3^	0–1000 μg/m^3^	About $25,000
		Met One Instrument Aerocet 831 Aerosol Mass Monitor [57]	Light Scatting	0.1 μg/m^3^	±10% of reading	0–1000 μg/m^3^	About $2000
		Alphasense OPC-N2 Particle Monitor [58]	Light Scatting	Not Provided	Not Provided	Not Provided	About $500
		Sharp Microelectronics DN7C3CA006 PM2.5 Module [59]	Light Obscuration (Nephelometer)	Not Provided	Not Provided	25–500 μg/m^3^	About $20
PM_10_		Teledyne Model 602 BetaPLUS Particle Measurement System [60]	Beta Attenuation	0.1 μg/m^3^	±1 μg/m^3^	0–1500 μg/m^3^	About $30,000
		Met One Instrument Aerocet 831 Aerosol Mass Monitor [57]	Light Scatting	0.1 μg/m^3^	±10% of reading	0–1000 μg/m^3^	About $2000
		Alphasense OPC-N2 Particle Monitor [58]	Light Scatting	Not Provided	Not Provided	Not Provided	About $500
		Sharp GP2Y1010AU Air Quality Sensor [61]	Light Obscuration (Nephelometer)	Not Provided	Not Provided	0–500 μg/m^3^	About $20
Lead (Pb)		Operation in Lab	-	-	-	-	-

**Table 6:** Instruments used in air quality monitoring systems (Part B).

Pollutant		Example Product	Measurement Method	Resolution	Accuracy	Range	Price (USD)
Carbon Monoxide (CO)		Teledyne Model T300U Gas Filter Correlation Carbon Monoxide Analyzer [62]	IR Absorption with Gas Filter Correlation Wheel	0.1 ppb	±0.5% of reading	0–100 ppb or 0–100 ppm	About $30,000
		Aeroqual Series 500 with CO Sensor Head [63]	Electrochemical Sensor	10 ppb	±0.5 ppm at 0–5 ppm or ±10% at 5–25 ppm	0–25 ppm	About $2000
		Alphasense B4 Series CO Sensor [64]	Electrochemical Sensor	4 ppb	Not Provided	0–1000 ppm	About $200
		Hanwei MQ-7 CO Sensor [65]	Solid-State Sensor	Not Provided	Not Provided	20–2000 ppm	About $10
Nitrogen Dioxide (NO_2_)		Teledyne Model T500U Nitrogen Dioxide Analyzer [66]	Cavity Attenuated Phase Shift Spectroscopy	0.1 ppb	±0.5% of reading	0–5 ppb or 0–1 ppm	About $30,000
		Aeroqual Series 500 with NO_2_ Sensor Head [67]	Electrochemical Sensor	1 ppb	±0.02 ppm at 0–0.2 ppm or ±10% at 0.2–1 ppm	0–1 ppm	About $2000
		Alphasense B4 Series NO_2_ Sensor [68]	Electrochemical Sensor	12 ppb	Not Provided	0–20 ppm	About $200
		SGXSensorTech MiCS-2714 NO_2_ Sensor [69]	Solid-State Sensor	Not Provided	Not Provided	0.05–10 ppm	About $10
Ozone (O_3_)		Teledyne Model 265E Chemiluminescence Ozone Analyzer [70]	Chemiluminescence Detection	0.1 ppb	±0.5% of reading	0–100 ppb or 0–2 ppm	About $25,000
		Aeroqual Series 500 with O_3_ Sensor Head [71]	Solid-State Sensor	1 ppb	±5 ppb	0–150 ppb	About $2000
		Alphasense B4 Series O_3_ Sensor [72]	Electrochemical Sensor	4 ppb	Not Provided	0–5 ppm	About $200
		Hanwei MQ-131 O_3_ Sensor [73]	Solid-State Sensor	Not Provided	Not Provided	10–1000 ppm	About $10
Sulfur Dioxide (SO_2_)		Teledyne Model 6400T/6400E Sulfur Dioxide Analyzer [74]	UV Fluorescence	0.1 ppb	±0.5% of reading	0–50 ppb or 0–20 ppm	About $30,000
		Aeroqual Series 500 with SO_2_ Sensor Head [75]	Electrochemical Sensor	10 ppb	±0.05 ppm at 0–0.5 ppm or ±10% at 0.5–10 ppm	0–10 ppm	About $2000
		Alphasense B4 Series SO_2_ Sensor [76]	Electrochemical Sensor	5 ppb	Not Provided	0–100 ppm	About $200
		Hanwei MQ-136 SO_2_ Sensor [77]	Solid-State Sensor	Not Provided	Not Provided	0–200 ppm	About $50

**Table 7:** Comparison of the five types of gas sensors.

Sensor Type	Detectable Gases	Linearity	Cross Sensitivity	Power Consumption	Maintenance	Response Time (T90)	Life Expectancy
Electro-chemical [78]	Gases which are electrochemically active, about 20 gases	Linear at room temperature	Can be eliminated by using chemical filter	Lowest, very little power consumption	Low	<50 s	1–2 years
Catalytic [79]	Combustible gases	Linear at 400 °C to 600 °C	No meaning when measuring mixed gases	Large, need to heat up to 400 °C to 600 °C	Lose sensitivity with time due to poisoning and burning out	<15 s	Up to 3 years
Solid-state [80]	About 150 different gases	Linear at operational temperature	Can be minimized by using appropriate filter	Large, need heating element to regulate temperature	Low	20 s to 90 s	10+ years
Non-dispersive Infrared [81]	Hydrocarbon gases and carbon dioxide	Nonlinear, need linearize procedure	All hydrocarbons share a similar absorption band, make them all cross sensitive	Small, mainly consume by the infrared source	The least	<20 s	3–5 years
Photo-ionization [82]	Volatile organic compounds (VOCs)	Relatively linear	Any VOCs with ionization potent- ials less than the ionizing potential of the lamp used will be measured	Medium, mainly consume by the ultraviolet source	The lamp requires frequent cleaning	<3 s	Depend on the Ultraviolet lamp, normally 6000 h

### 3.1. Gas Sensor

Nowadays, many different technologies for gas detection are available, each with certain advantages and disadvantages. To date, there are five types most suitable and widely used low-cost portable gas sensors, namely electrochemical sensors, catalytic sensors, solid-state (semiconductor) sensors, non-dispersive infrared radiation absorption (NDIR) and photo-ionization detector (PID) sensors (see Table 7). All of these sensors are low cost, light weight (less than one hundred grams) and with fast response time (in tenths seconds or few minutes). However, no single type of sensors is able to measure all the hazard gases (hundreds of hazard gases have been identified). Each type of sensors is sensitive to specific kinds of hazard gases.

Although, till now there is no low-cost portable gas sensor can achieve the same data accuracy and quality as conventional monitoring instruments. The low-cost portable gas sensors provide a fair enough accuracy and detection range [46]. What is more, all sensors need to be calibrated (When calibrating a sensor, the sensor is exposed to a specific kind of pollution gas with predefined concentration, the parameters of the sensor are adjusted such that the difference between the predefined gas concentration and the sensor output is minimized.) before operation and after a specific operational time. The necessity of calibration and the calibration procedures can be found in [83].

As described in Section 2, there are four types of hazard gases that we want to monitor most. They are carbon monoxide (CO), nitrogen dioxide (NO_2_), ground level ozone (O_3_) and sulfur dioxide (SO_2_). Combining the descriptions in [46,84] and the comparisons (with respect to sensor detectable gases, linearity, cross sensitivity, power consumption, maintenance, response time and life expectancy) in Table 7, two best types of sensors for these four types of hazard gases are determined.

- CO: Can be well detected by solid-state and electrochemical sensors.
- NO_2_: Can be well detected by solid-state and electrochemical sensors. Need to consider the interference gas O_3_. Proper methods can be applied to reduce the interference.
- O_3_: Can be well detected by solid-state and electrochemical sensors. Need to consider the interference gas NO_2_. Proper methods can be applied to reduce the interference.
- SO_2_: Can only be well detected by solid-state and electrochemical sensors. It poisons the catalytic sensors. The sensitivity of NDIR sensors is not high enough.

In a word, the solid-state and electrochemical sensors are the most suitable types of sensors to monitor these four types of hazard gases in building the TNGAPMS scenario. In fact, these two types of sensors are the basic elements in most of the existing works presented in Section 4. The operational principles of these two types of sensors are introduced as follows.

#### 3.1.1. Solid-state Gas Sensor [80]

The working principle of the solid-state ambient gas sensors was discovered when researchers were dealing with the semiconductor p-n junctions, which are sensitive to environmental gases.

**Figure 2:**
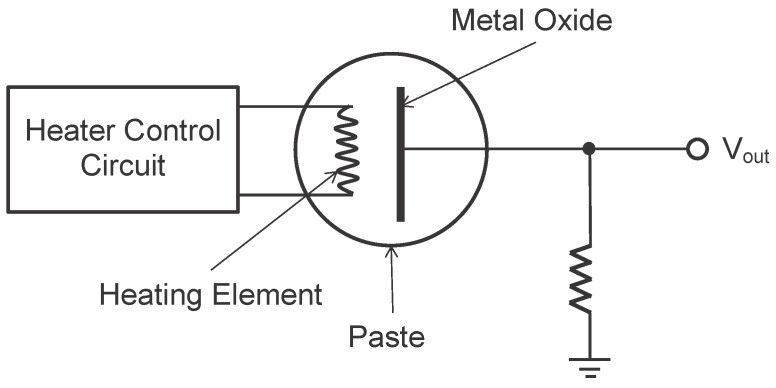
Bead-type sensor.

**Figure 3:**
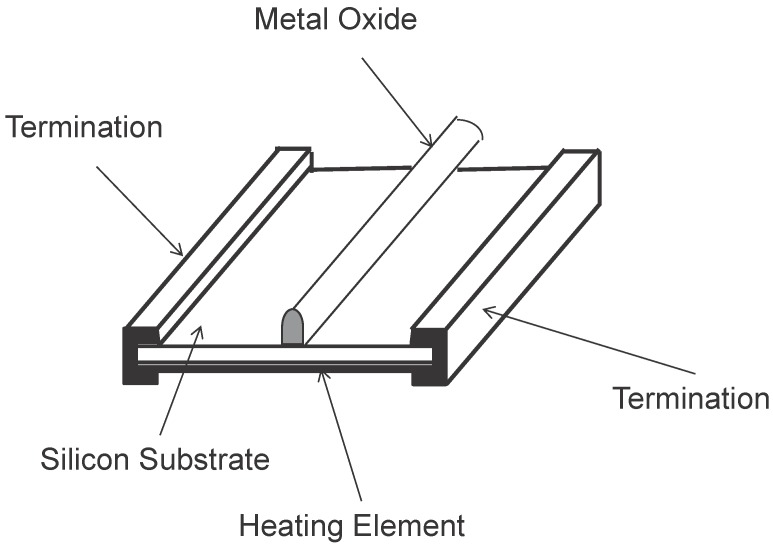
Chip-type Sensor.

A solid-state sensor consists of one or several metal oxides like tin oxide or aluminum oxide (the type of metal oxide being used depends on the target ambient gas the sensor aims for), and a heating element. The metal oxides can be processed into a paste, which is called bead-type sensor (see Figure 2). The metal oxides can also be deposited onto a silica chip similar to making semiconductors, which is called chip-type sensor (see Figure 3). When the metal oxides are exposed to the ambient gases, the gases will dissociate into charged ions or complexes that make the electrons accumulate on the surface of the metal oxides. The accumulation of electrons changes the conductivity of the metal oxides. By measuring the conductivity change, researchers are able to deduce the concentration of a specific kind of ambient gas.

In order to increase the reaction rate that results in a strong electrical signal, a heating element is used inside the solid-state ambient gas sensor. The heating element is also used to regular the temperature because the response (conductivity change) of a specific kind of ambient gas is different in different temperature ranges.

#### 3.1.2. Electrochemical Gas Sensor [78]

The working mechanisms of the electrochemical ambient gas sensors are electrochemical reactions (oxidation-reduction reactions, to be specific) within the sensors. The reaction between the sensor and the ambient gas molecules produces an electrical signal (current) proportional to the concentration of the ambient gas.

**Figure 4:**
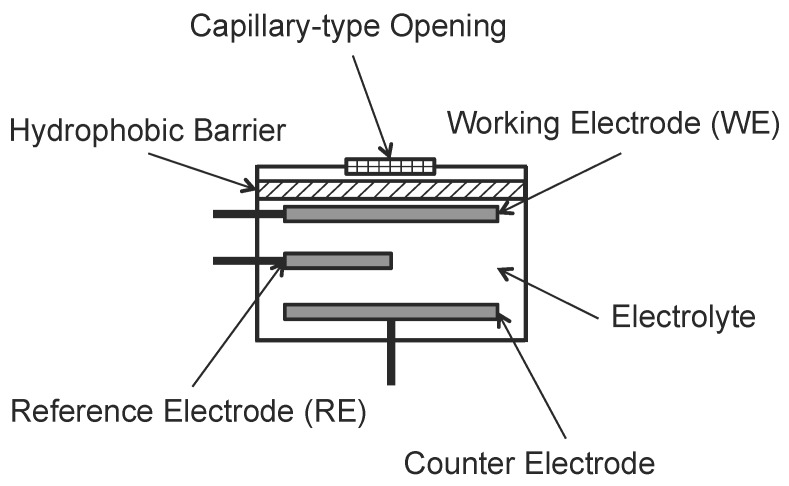
Basic Electrochemical Sensor.

An electrochemical sensor consists of a Working Electrode (WE) and a Counter Electrode (CE). For sensors requiring an external driving voltage, a Reference Electrode (RE) is needed. These two or three electrodes are separately deployed into the electrolyte within the sensor (see Figure 4).

Different sensors may use different types of selective membranes, electrolyte and working electrodes in order to improve the sensor’s selectivity to a specific kind of ambient gas. To allow enough amount of ambient gas to react with the sensor while preventing electrolyte leakage, the ambient gas first goes through a capillary-type opening and a hydrophobic barrier. When the ambient gas reaches the working electrode, the oxidation-reduction reaction occurs. The specifically developed electrode for an interested ambient gas catalyzes these reactions. By measuring the current between the Working Electrode (WE) and the Counter Electrode (CE), researchers are able to deduce the concentration of the target ambient gas. For sensor with Reference Electrode (RE), the reference electrode is used to control the oxidation and reduction reactions and reduce the potential drift on working electrode due to deterioration (may not work when the electrodes are fouled).

Note that, most of the electrochemical ambient gas sensors require a small amount of oxygen and humidity to function properly. Also, wind velocity influences the chemical equilibrium on the sensor’s surface and further influences the sensor’s readings [85].

### 3.2. Particulate Matter Sensor

The measurement of particulate matter (PM) is not straightforward and there are many techniques (used in conventional monitoring systems and TNGAPMSs) available for measuring the mass concentrations of PM. Due to the complex nature of PM, different measurement techniques may give different results [86]. Some conventional monitoring instruments use a heating element to eliminate the effect of changing humidity and temperature. However, the heating element evaporates the semi-volatile species and influences the measurement results. Therefore, some instruments use a special dryer instead of a heating element (e.g., the Nafion dryer [87]).

The available techniques for measuring the concentration of PM can be grouped into two categories. One is direct reading instrument which provides continuous measurements (sampling interval is in seconds or minutes) on the concentration of PM in ambient air (see Table 8). The other one is filters-based gravimetric sampler, which collects the PM onto a filter that needs to be weighted periodically in lab. The weighting procedure is a time and human resources consuming task, which leads to a large delay (in days) between collection and reporting. However, the filters-based gravimetric technique is usually used as the reference method in government agencies. One should note that the reference methods are not the absolute methods but subject to many artifacts (temperature and humidity change and semi-volatile compounds).

The commonly used continuous measurement techniques of PM in ambient air are listed as follows.

#### 3.2.1. Tapered Element Oscillating Micro-Balance (TEOM) Analyzers [88]

The TEOM analyzers are widely used in the conventional air pollution monitoring systems. The operation principle of TEOM is that the oscillation frequency of the tapered glass tube is proportional to the mass of the tube. The PMs deposited onto the tube will change the mass and oscillation frequency of the tube. By measuring the oscillation frequency change of the tube and the volume of air sampled, researchers are able to deduce the mass concentration (μg/m^3^) of PM in ambient air.

Note that the air is sampled through a size selective inlet. For example, a PM_10_ size selective inlet rejects 50% (no design can reject 100%) of the particles with diameter more than 10 μm and let through particles with diameter of 10 μm and less. In order to eliminate the effect of humidity change, a heating element or a dryer is used.

#### 3.2.2. *β*-Attenuation Analyzers [89]

The *β*-Attenuation Analyzers or *β*-Attenuation Monitors (BAM) are the most widely used PM measurement instruments in the conventional air pollution monitoring systems. The air is first sampled through a size selective inlet (PM_10_ or PM_2.5_) with or without heater/dryer that minimizes the water contained in the air. Then the air goes through a paper filter, which catches the PM. The paper filter with PM is exposed to *β*-attenuation source. After the measurement interval, researchers are able to deduce the mass of the PM on the filter by measuring the radiation intensity of the filter.

#### 3.2.3. Black Smoke Method [90]

The black smoke technique collects the PM on a paper filter over 24 h period through a size selective inlet. The darkness of the paper filter is then measured by a reflectometer and converted to the PM’s mass concentration. This kind of monitoring equipment is relative simple, robust and cost-efficient. However, the mass concentration is derived by measuring the darkness of the filter and the darkness of PM varies in different locations. This makes the darkness-to-mass coefficient change from time to time and location to location.

#### 3.2.4. Optical Analyzers [91]

The optical analyzers utilize the interaction between the ambient PM and the imaging, laser or infrared light. These analyzers are small, lightweight and battery operated. Base on the optical principle, the optical analyzers can be classified into three categories, namely direct imaging, light scatting and light obscuration (nephalometer) analyzers.

**Figure 5:**
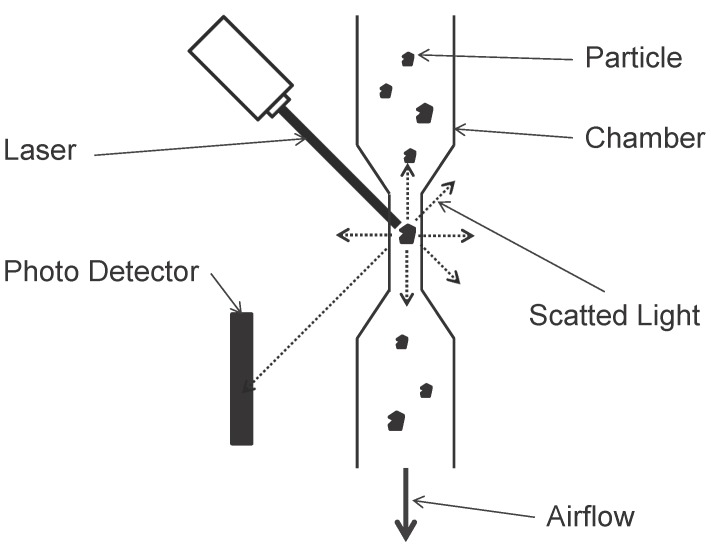
Basic Light Scatting Particle Counter.

**Figure 6:**
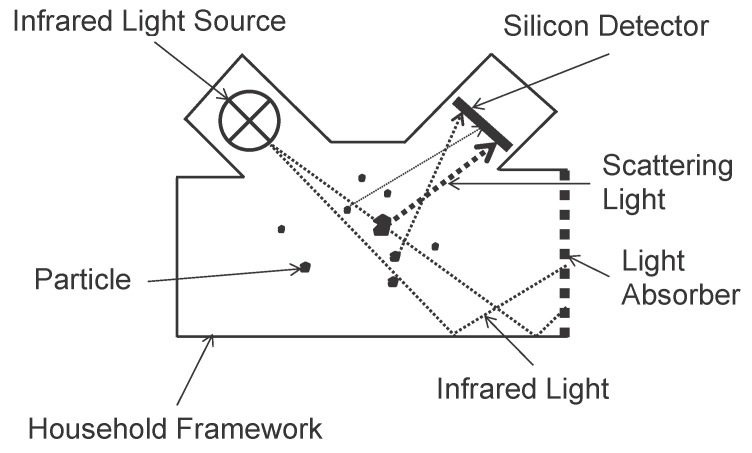
Basic Nephelometer.

- Light Scatting:
  This category of optical analyzers uses a high-energy laser as the light source. When a particle passes through the detection chamber that only allows single particle sampling, the laser light is scattered by the particle. A photo detector detects the scatting light. By analyzing the intensity of the scatting light, researchers can deduce the size of the particle. Also, the number of particle counts can be deduced by counting the number of detecting light on the photo detector (see Figure 5). The advantage of this approach is that a single analyzer can detect particles with different diameters simultaneously (*i.e.*, PM_2.5_, PM_5_ and PM_10_). However, the particle counts need to be converted to mass concentration by calculation (depends on the particle counts, particle types and particle shapes) and this will introduce errors that further affect the precision and accuracy of the analyzers.
- Direct Imaging:
  In a direct imaging particle analyzer, a beam of halogen light illuminates the particles and the shadow of each particle is projected to a high definition, high magnification and high resolution camera. The camera records the passing particles. The video is then analyzed by computer software to measure the PM’s attributes. Both size and counts of the PMs in the ambient air can be obtained. What’s more, the color and the shape of the particles can also be detected.
- Light Obscuration (Nephelometer):
  This category of optical analyzers uses the fastest particle concentration (μg/m^3^) measurement method with high precision and low detection limited. A nephelometer is an instrument that measures the size and mass concentration of PM in the ambient air. In a nephelometer, a near infrared LED is used as the light source and a silicon detector is used to measure the total light scattered (which is majorly responsible for the total light extinction) by the PMs (see Figure 6). By analyzing the intensities (in magnitude) of the scattered light and the shape of the scattering pattern, both the size distribution and the mass concentration can be determined right away [92].

The comparisons of these four types of PM measuring techniques are shown in Table 8. Because of the high data resolution and accuracy, large size, heavy weight and high cost, the TEOMs and BAMs are typically used in the conventional air pollution monitoring systems. Although the readings from the light scatting and the light obscuration optical analyzers are with relative low resolution and accuracy, and the particle-count-to-mass-concentration coefficient is different from time to time and location to location, these two types of PM sensors are widely used in hand-held monitoring devices and TNGAPMSs because of their small size, light weight, low cost and simultaneously measuring ability.

**Table 8:** Comparison of four types of particulate matter (PM) measurement methods.

Measurement Method	Advantages	Disadvantages	Accuracy
Tapered Element Oscillating Microbalance (TEOM) analyzers	Provide real time (<1 h) data with high precision.	A heater must be used which leads to lose of semi-volatile material. Usually with large size, heavy weight and high cost.	±0.5 μg/m^3^
*β*-attenuation analyzers (BAM)	Provide real time (<1 h) data with high precision.	A radioactive source is used. If heater is used some semi-volatile material may be lost. Need to replace the paper filter periodically. Usually with large size, heavy weight and high cost.	±1.0 μg/m^3^
Black smoke method	Simple, robust and inexpensive. Easy to maintain. Short sample time (in minutes).	Measure the darkness rather than the mass concentration of the particulate matters. Darkness-mass factor may change from time to time and location to location.	±2.0 μg/m^3^, or higher
Optical analyzers	Small, light weight and usually battery operated. Short sample time (in seconds or minutes). Can measure different sizes of particles simultaneously.	Depends on some assumptions of particle characteristics (e.g. each particle is perfect bean-like shape). These assumptions may be different from time to time and location to location.	Depends on the analyzer type and usually not specifically declared by the manufacture.

## 4. State-of-The-Art WSN Based Air Pollution Monitoring Systems

Twenty state-of-the-art TNGAPMSs that significantly improve the spatio-temporal resolution of the air pollution information and the quality of services provided are presented in this section. The existing works are classified into three categories based on the carriers of the sensor nodes, and the advantages and disadvantages of each category are discussed.

Air pollution in urban areas with ubiquitous emission sources attracts extensive attentions worldwide due to the tremendous impacts on human lives at anytime and anywhere. Networks of monitoring stations using traditional measurement instruments have been deployed to mitigate these impacts. Data acquired by these stations can be utilized for building pollution maps and models that provide authorized environmental situation information and prediction. However, limitations in spatio-temporal resolution and Quality of Services (QoS) are prevalent in these systems [93,94,95]. These limitations result in issues and problems of the conventional air pollution monitoring systems, like non-scalability of system, limited data availability on personal exposure, and out-of-the-fact warnings on acute exposure.

In order to address these prevalent problems, researchers have put lots of efforts into the concept of TNGAPMS by utilizing the advance sensing techniques, MicroElectroMechanical Systems (MEMS), and Wireless Sensor Networks (WSN).

According to the definition of participatory sensing [96,97] and vehicular wireless sensor networks [98,99], and our insights while reviewing the related works, the existing works are classified into three categories based on the carriers of the sensor nodes, namely Static Sensor Network (SSN. Sensor nodes are usually mounted on the streetlight or traffic light poles, or carefully selected locations.), Community Sensor Network (CSN. Sensor nodes are carried by the public communities, usually by volunteers or people who are keen on air quality.), and Vehicle Sensor Network (VSN. Sensor nodes are carried by the public transportations or specially equipped cars.).

**Figure 7:**
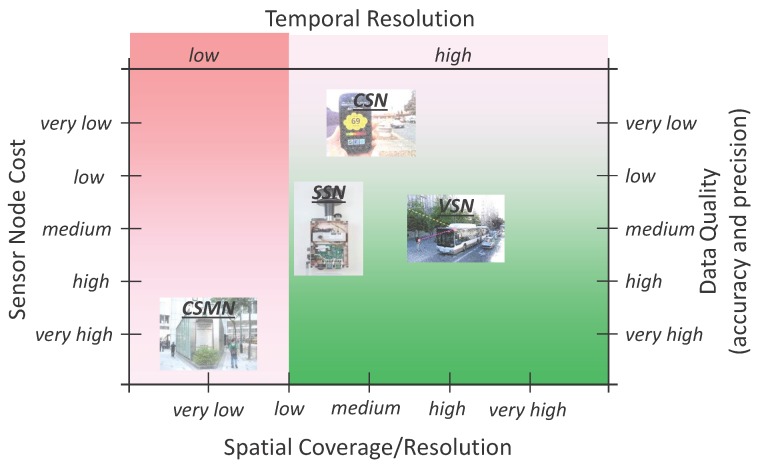
Trade-off between tolerable sensor node cost, obtainable measurement coverage/resolution, expected data quality and achievable measurement temporal resolution for Conventional Stationary Monitoring Network (CSMN), Static Sensor Network (SSN), Community Sensor Network (CSN) and Vehicle Sensor Network (VSN) [100].

These existing works greatly improve the spatio-temporal resolution and QoS of the air pollution information compared with that of conventional monitoring systems. However, in TNGAPMSs, it is impossible to use the same high-end measurement instruments as the ones utilized in stationary monitors of Conventional Stationary Monitoring Network (CSMN). Hence, whenever we deal with the TNGAPMSs, we face the same interesting trade-off as shown in Figure 7. In the following subsections, the three types of sensor networks (SSN, CSN and VSN) are discussed in detail.

### 4.1. Static Sensor Network (SSN)

**Figure 8:**
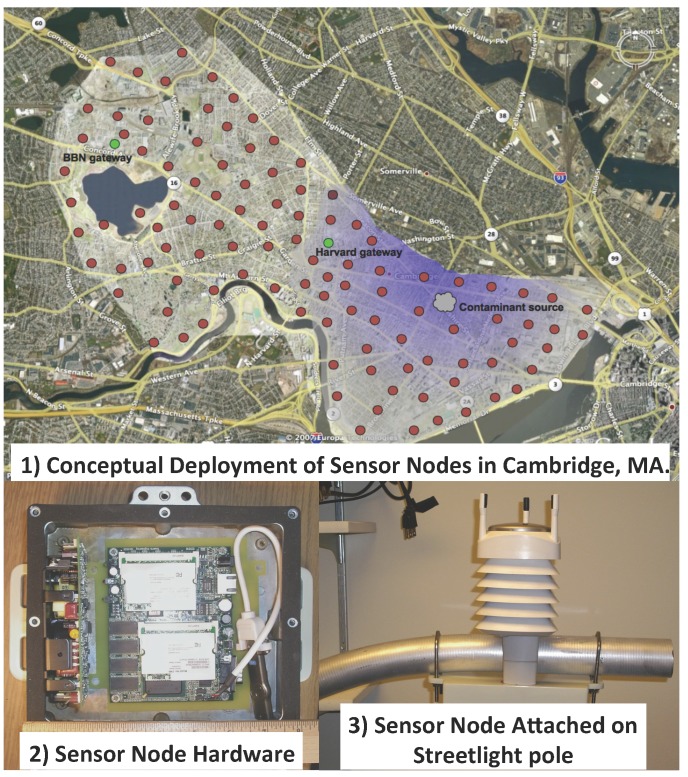
Example of the SSN system architecture and prototype. Red dots are the sensor nodes. Green dots are the gateways that forwarding the acquired data to the Contaminant Source. Figures are adapted from [101].

In SSN systems, the sensor nodes are typically mounted on the streetlight or traffic light poles, or walls (see Figure 8). By utilizing the low-cost ambient sensors, the number of sensor nodes in SSN systems is much larger than that in the conventional monitoring systems. Air pollution information with high spatio-temporal resolution is achievable in SSN systems. Authorized air pollution information is available to the public through web pages, Web Apps, mobile Apps, *etc*.

***Carrier:*** The sensor nodes are usually mounted on the streetlight or traffic light poles, or carefully selected locations.

**Related Works:**

In [101], the project CitySense was presented. This paper claimed that most research groups of WSN evaluate their ideas by simulations, small-scale test deployments or large-scale test deployments with narrow range of target environment, which may have potential issues and problems in real-life large-scale applications. The motivations of the CitySense project are to provide an urban-scale wireless networking testbed, which is able to support a wide range of applications including outdoor air pollution monitoring. Each sensor node consists of a Linux PC, dual 802.11 a/b/g radios and a wide range of sensors. These sensors nodes are mounted on and powered by the streetlights. Sensing data are uploaded to the server through Wi-Fi and authorized air pollution information is available to the public through a customized Web App.

In [102], a WSN based urban air quality monitoring system was proposed. This system consists of a set of sensor nodes, a gateway and a centralize control system provided by the LabVIEW program. Each sensor node integrates with a ZigBee communication link, a CO sensor and a battery. And the gateway is consisted of a Global System for Mobile (GSM) communication link and a wind speed and direction sensor. Data from the sensor nodes are uploaded to the gateway and further forwarded to the central system. This system was deployed to the main roads in Taipei city and the experiment results illustrated that the system can provide micro-scale air pollution information in real-time.

In [103], an outdoor ambient real-time air quality monitoring system was proposed, implemented and tested. In this system, the concentration of O_3_, NO_2_, CO and H_2_S are sensed and transmitted back to the backend server through the GPRS wireless communication link every minute. Authorized air pollution information is available to the public through the customized Web and mobile Apps. A solar panel was utilized to solve the power constraint issue of the sensor nodes (stationary).

In [104], an innovative system named Wireless Sensor Network Air Pollution Monitoring System (WAPMS) was proposed and simulated to monitor the outdoor air pollution situations in Mauritius. This system comprises of an array of sensor nodes and a communication system that gathers the air pollution data to the server. The air pollution data are acquired and passed to the cluster heads by the sensor nodes autonomously. The cluster heads then forward the data to the server. In order to minimize the power consumption in the WSN, a novel data aggregation algorithm named Recursive Converging Quartiles (RCQ) was proposed and implemented. Moreover, a hierarchical routing protocol was utilized to maximize the sensor nodes’ energy efficiency.

In [105], an outdoor WSN based air quality monitoring system (WSNAQMS) for industrial and urban areas was proposed. The sensor node consists of a set of gas sensors (O_3_, CO and NO_2_) and a ZigBee wireless communication link based on the Libelium’s [106] gas sensing capable mote. Data are uploaded to the central server through the ZigBee communication link. Authorized air pollution information is available to the public through Email, SMS and customized Web App. This framework is claimed to be simple and reusable in other applications. Also the failure sensor nodes can be identified efficiently and the energy consumption of each sensor node is minimized. Moreover, a simple Clustering Protocol of Air Sensor (CPAS) network was proposed, which proved to be efficient (in simulation) in terms of network energy consumption, network lifetime, and the data communication rate. The QoSs of the network such as delay, accuracy and reliability (fault tolerance) were also considered.

In [107], a WSN based indoor air pollution monitoring system was presented. The focuses were the power consumption on sensor, sensor node and network levels. Several methodologies that greatly improved the lifetime (up to 3 years) of the monitoring system have been proposed and simulated. The sensor node equips with several sensors (accelerometer, temperature and relative humidity sensors, CO, VOCs and motion sensors), a ZigBee communication link and a battery. In the simulation, 36 sensor nodes were place in the first floor of a 4-story building. Data acquired by the sensor nodes were available to the researchers only.

In [108], an indoor and an outdoor air pollution monitoring architectures based on Wi-Fi were proposed. In this paper, only the indoor one was implemented and tested. Each sensor node consists of several sensors (temperature and relative humidity sensors, CO, methane and solvent vapors sensors) and a Wi-Fi communication link. In order to mitigate the influence factors (temperature and relative humidity) of the gas sensors, a neural network was implemented to obtain the temperature and relative humidity correcting values for the pollutants’ concentrations. Sensed data were processed by a PC and published to a customized web page.

***Adavantages:***

1. Loose constraint on energy consumption (The sensor nodes are typically powered by batteries with large capacity or energy harvest devices or power line.)
2. No locating device (The location of a sensor node is known once it was deployed since the sensor node is stationary.)
3. Loose limitations on weight and size (The carrier of the sensor node is able to carry sufficient enough weight.)
4. Multiple sensors per node (One sensor node can equip with several types of sensors because of the loose limitations on weight and size.)
5. Accurate and reliable data (Sensor node can integrate with assisting tools because of the loose limitations on weight and size.)
6. Guaranteed network connectivity (Once the stationary sensor node joined the network, the topology is fixed and the connectivity is guaranteed.)
7. Well calibrated and maintained sensors (The sensor nodes can be well calibrated and maintained by the professionals periodically.)

***Disadvantages or Challenges:***

1. Careful placement of sensor nodes requirement (This is because of the location dependence of air pollutants.)
2. Large number of sensor nodes requirement (Data with sufficient geographic coverage and spatial resolution are only achievable by increasing the number of the stationary sensor nodes.)
3. Resource wasting in certain level (The stationary sensor nodes are in sleep mode most of the time because continuously updating data at one location is pointless [13].)
4. Inconveniences of calibration and maintenance (The professionals need to visit all stationary sensor nodes, which is a time and manpower consuming task, to perform operations.)
5. 2-Dimensional data acquisition (Only the air quality of urban surface is monitored.)
6. Customized network requirement (A customized wireless or wired network is required when the cellular network is not utilized.)

### 4.2. Community Sensor Network (CSN)

**Figure 9:**
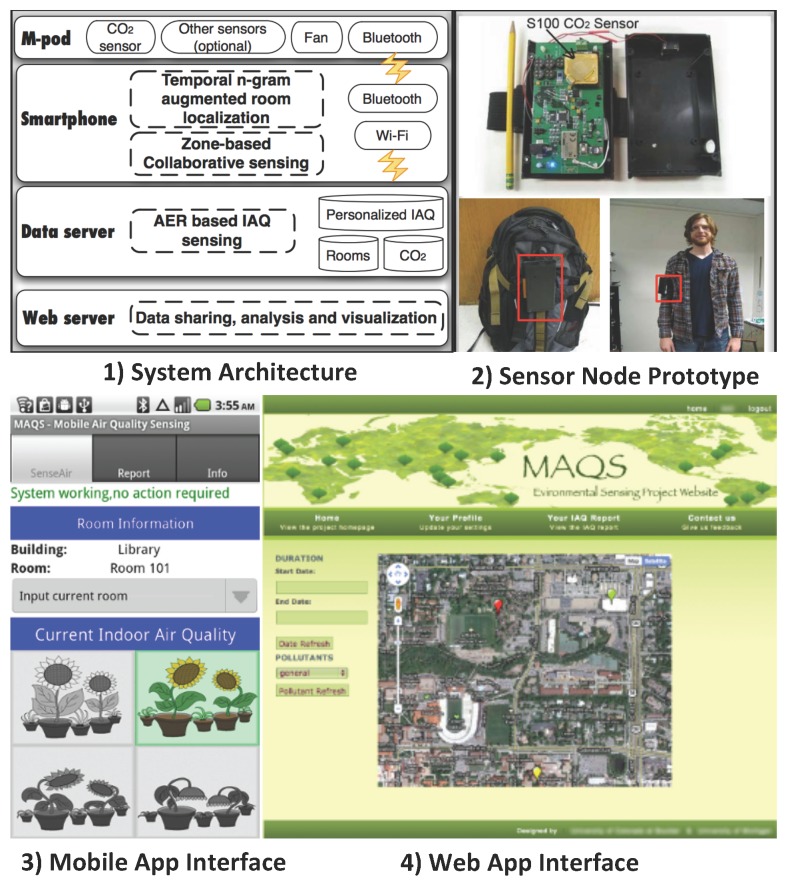
Example of the Community Sensor Network (CSN) system architecture and prototype. Figures are adapted from [109].

In CSN (or Participatory Sensing) systems, the sensor nodes are typically carried by the users (see Figure 9). By utilizing the low-cost portable ambient sensors and the ubiquitous smart phones, users are able to acquire, analyze and share the local air pollution information [96]. Authorized air pollution information is available to the public through web pages, Web Apps, mobile Apps, *etc.*

***Carrier:*** The sensor nodes are carried by the public or professional users, usually by volunteers or people who are keen on air quality.

**Related Works:**

In [10], a low-power and low-cost mobile sensing system for outdoor participatory air pollution monitoring called GasMobile was introduced. The sensor node composes of a small-size, low-cost O_3_ sensor and an off-the-shelf smart phone. The sensor communicates with the smart phone through the USB port. Data (tagged with location information from the build-in GPS module) are uploaded to the server through the cellular network. Authorized information is available to the public through the customized Web and mobile Apps. Two methods were proposed and implemented to improve the data quality of the sensor. This paper claimed that air pollution information with high spatial resolution can be achieved by the community-driven sensing infrastructure like OpenSense [110].

In [97], an outdoor air quality sensing system (P-Sense) based on the participatory sensing technology was presented. Each sensor node consists of a set of sensors (CO_2_, CO, VOCs, H_2_, temperature and relative humidity) and a Bluetooth link. Data are acquired by the sensors and transmitted to the smart phone through the Bluetooth link. The smart phone then uploaded the data to the server through the cellular network. Authorized air pollution information is available to the public through the customized Web and mobile Apps. Several research issues that need to be addressed before practical deployment of the P-sense system were also pointed out.

In [109], a personalized mobile indoor air quality sensing system called MAQS was presented. Each sensor node consists of several sensors (CO_2_, CO, O_3_, temperature and relative humidity sensors) and a Bluetooth link communicating with the smart phone. The smart phone further forwards the data to the server using a build-in Wi-Fi module, which was also utilized for localization. Authorized air pollution information is available to the public through the customized Web and mobile Apps. Three novel techniques were proposed and implemented to improve the data accuracy and energy efficiency of the system.

In [111], a hardware and software platform for outdoor participatory air quality monitoring, called N-SMART was introduced. By attaching sensors (CO, NO_X_, temperature and Bluetooth) to a GPS-enabled cellphone, the raw air pollution data, which help understand the impacts of air pollution on both individuals and communities, are gathered. The sensor node communicates with the cellphone through the Bluetooth wireless link. Note that, this paper didn’t focus on the implementation but the design of the sensing platform. Several research challenges like unpredictable user behaviors and movements, and user privacy problems were discussed in this paper.

In [112], an outdoor urban noise pollution monitoring system called NoiseTube was proposed and implemented. Although it is not an urban air pollution monitoring system, the system architecture and implementation are very similar. Each sensor node is the smart phone itself. The noise data (tagged with location information from the build-in GPS module) are collected by the build-in microphone. Collected data are uploaded to the server through the cellular network. Authorized noise pollution information is available to the public through the customized web page and mobile App.

In [113], a Volatile Organic Compounds (VOCs) sensor node with high selectivity and sensitivity was developed. The authors focused more on the development of the novel tuning fork sensors than the implementation of the air quality monitoring system. Each sensor node consists of several tuning fork sensors (detecting VOCs, temperature and relative humidity) and a Bluetooth device communicating with the smart phone. A customized mobile App for visualizing the sensing data was implemented.

***Adavantages:***

1. Cost efficiency (The sensor node utilizes the cellphone’s GPS module and the cellular network, or even the cellphone’s computational power.)
2. Coupled data generators and consumers (Local or personal air pollution information is available.)
3. Public-driven property (The cost of the sensor nodes and the data transmission can be apportioned by the users. It is costly and infeasible for a single agency to acquire all the sensor nodes.)
4. Automatic gathering property (The sensor nodes are densely distributed at locations with gathering people automatically. Data with higher spatial resolution and accuracy are achievable in such case.)
5. Mobility of sensor nodes (The mobility of the cellphones or users enlarges the sensor node’s geographic coverage.)
6. Public behaviors acquisition ability (Information such as the public movement patterns, and interaction between air quality and public behaviors, is achievable.)

***Disadvantages or Challenges:***

1. Low data accuracy and reliability (The sensor nodes are typically put in pockets or handbags. Also, the users spend significant amount of time indoor or inside cars [114]).
2. Privacy issues (The users may not want to make their location information public for privacy issues).
3. Badly calibrated and maintained sensors (Professional calibrations of sensors performed by the public users are very unlikely. Frequent collections and calibrations of sensors by the professionals are infeasible).
4. Serious constraint on energy consumption (The sensor nodes is typically powered by cellphone’s battery or battery with small capacity).
5. Uncontrolled or semi-controlled mobility (The routes of the sensor nodes or users are pre-determined. The sensor nodes may squeeze into a small place with crowded people and cause redundant sampling. Some locations may never be visited).
6. 2-Dimensional data acquisition (Only the air quality of urban surface is monitored).
7. Serious limitations on weight and size (The sensor node should be portable, which affects the accuracy, reliability and number of sensors equipped, because it is carried by user).

### 4.3. Vehicle Sensor Network (VSN)

**Figure 10:**
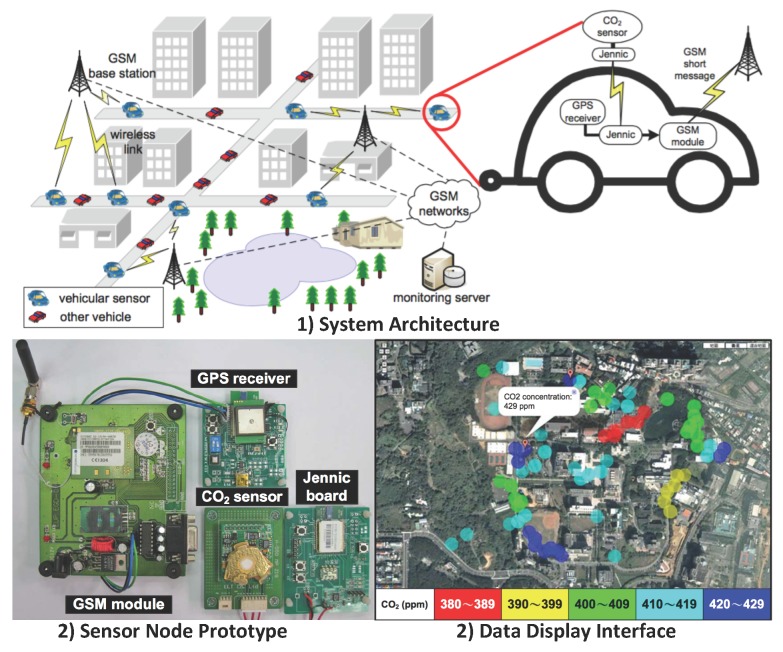
Example of the Vehicle Sensor Network (VSN) system architecture and prototype. Figures are adapted from [98].

In VSN systems, the sensor nodes are typically carried by the public transportations like buses or taxis (see Figure 10). By utilizing the low-cost portable ambient sensors and the mobility of vehicles, one sensor node is able to achieve sufficient large geographic coverage [99,115]. Authorized air pollution information is available to the public through web pages, Web Apps, mobile Apps, *etc.*

***Carrier:*** The sensor nodes are carried by the public transportations (buses, trains and taxis) or specially equipped cars.

**Related Works:**

In [11], the Mobile Air Quality Monitoring Network (MAQUMON) was presented. This system is composed by a number of car-mounted sensor nodes measuring the concentrations of O_3_, CO and NO_2_. Each sensor node utilizes a GPS module for acquiring time and location information and a Bluetooth link for communicating with the laptop inside car. Collected data are then uploaded to the server through the laptop’s Wi-Fi link. Authorized air pollution information is accessible through the sensor node’s LCD display or the SensorMap Web App.

In [19], a distributed infrastructure based on the WSN and Grid computing for real-time comprehensive air pollution monitoring and mining was presented. In this system, two types of sensor nodes are utilized, namely the Mobile Sensor Node (MS node) and the Static Sensor Node (SS node). The sensor node consists of a Generic Ultra Violet Sensor Technologies and Observation (GUSTO) sensor (able to detect SO_2_, NO_X_, O_3_ and VOCs) and a wireless link (ZigBee or Wi-Fi or others). The MS nodes are mounted on the public transportations and transmitting data to the SS nodes. The SS nodes are able to perform data acquisition and fusion, and further forward the data (from MS nodes and SS nodes) to the central server. Currently, the air pollution information is only available to the researchers. A distributed data mining-algorithm for identifying the relationships between the urban transport and the environment was also proposed.

In [98,116], a vehicular wireless sensor network architecture was proposed and implemented to achieve the micro-climate monitoring. A CO_2_ sensor is mounted outside the car to monitor the concentration of CO_2_. A ZigBee intra-vehicle wireless network is utilized to communicate with the inside-car processing unit, which equipped with a micro-controller, a GSM short message module and a GPS module. Data are then sent to the GSM base stations and further forwarded to the monitoring server. Authorized air pollution information is available to the public through a Web App. In order to balance the accuracy of sensed data and the cost of communication, an on-demand approach that adjusts vehicles’ reporting rates was proposed.

In [99], a mobile sensor node prototype that can be mounted on vehicle was introduced and tested. Each sensor node consists of a set of sensors (CO, PM, NO, NO_2_ and VOCs) for detecting the pollutants’ concentrations, a GPS module for collecting the location information, and a GPRS or Wi-Fi module for communicating with the server. Analyzed data are available to the public through a Web App. This paper claimed that the proposed system demonstrated higher spatial coverage at the expense of lower temporal resolution compared with the SSN systems.

In [115], a low-cost air pollution monitoring system using vehicular sensor network was proposed to complement the conventional air pollution monitoring networks. Each sensor node consists of a set of sensors (temperature, relative humidity, NO_2_, CO_2_, CO and O_3_), a GPS module, and a ZigBee wireless link. Data acquired by the sensor nodes are transmitted back to the central computer for further analysis through the static ZigBee accessing points. These sensor nodes are mounted on the public transportations, like buses. By utilizing the mobility of the public transportations, even with a few sensor nodes, the urban air pollution information with fine-grained (high spatial resolution) level was achieved. In this paper, the air pollution information is only available to the researchers.

In [117], a fine-grained vehicular-based mobile air pollution measuring approach was presented. The proposed schema can utilize multiple types of mobile sensor nodes including the proposed Mobile Sensing Box (MSB) and other personal sensing devices. The MSB consists of two ambient sensors (CO and PM) for data collection, a GPS module for location and time information acquisition and a cellular module for data transmission. The car mounted with a MSB travels around the city. Real-time data are received and analyzed by the Cloud Server. Authorized air pollution information is available to the public through the customized Web and mobile Apps.

In [118], a GPRS Sensor Array for outdoor air pollution monitoring was proposed, implemented and tested. The system consists of a mobile sensing unit, which was mounted on the public transportation, and an Internet enabled server. Each sensing unit integrated with a set of sensors (CO, NO_2_ and SO_2_), a GPS and GPRS modules. Data with location information are sent to the server through the cellular network (GPRS) for further processing and analysis. Authorized air pollution information is available to the public through the customized Web App.

***Adavantages:***

1. Loose constraint on energy consumption (The sensor nodes are powered by the vehicles’ batteries.)
2. Loose limitations on weight and size (The carrier of the sensor node is able to carry sufficient enough weight.)
3. Multiple sensors per node (One sensor node can equip with several types of sensors because of the loose limitations on weight and size.)
4. Accurate and reliable data (Sensor node can integrate with assisting tools because of the loose limitations on weight and size.)
5. High mobility of sensor nodes (The highly mobile vehicles significantly enlarge the sensor node’s geographic coverage.)
6. Feasibility in maintenance (The vehicles mounted with sensor nodes can be driven to a specific location. Professionals can perform maintenance on large amount of sensor nodes simultaneously.)
7. Well calibrated and maintained sensors (This is because of the feasibility in maintenance of the VSN systems.)
8. Automatic gathering property (The sensor nodes are densely distributed at locations with gathering public transportations automatically. Data with higher spatial resolution and accuracy are achievable in such case.)

***Disadvantages or Challenges:***

1. Uncontrolled or semi-controlled mobility (The routes of the sensor nodes or public transportations are pre-determined. The sensor nodes may squeeze into a small place with crowded transportations and cause redundant sampling. Some locations may never be visited.)
2. Redundant sampling issues (The vehicles may be trapped into traffic jams or parked in parking lots that cause redundant sampling. This issue compromises the spatial and temporal resolutions.)
3. Cost inefficiency on carriers (The specially equipped cars may cost a huge amount of money.)
4. Locating and communication devices requirement (The system requires GPS modules, and wireless modules or cellular modules.)
5. Customized network requirement (A customized wireless network is required when the cellular network is not utilized. The network connectivity may not be guaranteed due to the mobility of vehicles.)
6. 2-Dimensional data acquisition (Only the air quality of urban surface is monitored.)
7. Spatial-to-Temporal resolution trade-off (Higher spatial coverage at the expense of lower temporal resolution [99].)

In this section, 20 state-of-the-art TNGAPMSs are discussed and classified into three categories, namely the SSN, CSN and VSN. Summary information (with respect to the Carrier, WSN Type, Sensor Type, Power Source, Locating Device, Computational Power, Operation Environment, Sensing Periodic, Number of Sensor Nodes in System, Geographic Coverage and Data Availability) of these systems is shown in Table 9 and Table 10. Although these systems greatly improve the pollution information’s spatio-temporal resolution compared with the conventional monitoring systems, there exist some issues or challenges in these TNGAPMSs that we will discuss in Section 6.

**Table 9:** Summary information of the 20 systems in literature works (Part A) (* means unknown).

Sensor Network Type	System	Carrier	WSN Type	Sensor Type	Power Source	Locating Device	Computational Power of Sensor Node (Clock Speed/SRAM/Storage)
SSN	In [105]	Not mentioned	ZigBee	Electrochemical (O_3_, CO, NO_2_)	Not mentioned	None	Arduino (14 MHz/512 KB/2 GB)
	In [101]	Streetlight pole	Wi-Fi (802.11 a/b/g)	Solid-state (CO_2_, NO, O_3_)	Power line	None	Linux based embedded PC (266 MHz/128 MB/1 GB)
	In [104]	Not mentioned	Not mentioned	Not mentioned	Not mentioned	Not mentioned	Not mentioned
	In [102]	Streetlight pole	ZigBee + Cellular network (GSM)	Solid-state (CO)	Battery	None	Octopus II (1 MHz/10 KB/1 MB)
	In [108]	Wall	Wi-Fi (802.11 b/g)	Solid-state (CO, VOCs)	Not mentioned	None	IPu8930 (*/*/512KB)
	In [107]	Wall	ZigBee	Solid-state (CO, VOCs)	Battery	None	JN5168 (32MIPs/128KB/*)
	In [103]	Station	Cellular network (GPRS)	Solid-state (CO, NO_2_, O_3_, H_2_S)	Battery, Solar panel	None	Arduino (16 MHz/8 KB/2 GB)
CSN	In [10]	Public user	Cellular network	Solid-state (O_3_)	Battery	Cellphone GPS module	HTC HERO saxophone
	In [111]	Public user	Not mentioned	Not mentioned	Cellphone battery	Cellphone GPS module	LG VX980 smart phone
	In [109]	Public user	Wi-Fi	NDIR (CO_2_), Solid-state (CO, O_3_)	Battery	Cellphone Wi-Fi module	Arduino (16 MHz/2 KB/32 KB)
	In [112]	Public user	Cellular network	Microphone	Cellphone battery	Cellphone GPS module	NOKIA N95 cellphone
	In [97]	Public user	Cellular network	Solid-state (CO_2_, VOCs), Catalytic (H_2_), Electrochemical (CO)	Battery	Cellphone GPS module	PRO200 Sanyo cellphone
	In [113]	Public user	Cellular network	QTF (VOCs)	Battery	Cellphone GPS module	Motorola Q phone
VSN	In [115]	Public transportation	ZigBee	Solid-state (CO, NO_2_, O_3_, CO_2_)	Bus battery	GPS module	Arduino (16 MHz/8 KB/*)
	In [11]	Car	Wi-Fi	Solid-state (CO, NO_2_, O_3_)	Battery	GPS module	8051 uC (*/4KB/2MB)
	In [98]	Car	Cellular network (GSM)	NDIR (CO_2_)	Car battery	GPS module	JN5139 (16 MHz/96 KB/192 KB)
	In [117]	Car	Cellular network	Solid-state (CO), Optical analyzer (PM)	Bus battery	GPS module	Arduino (16 MHz/8 KB/128 KB)
	In [118]	Bus	Cellular network (GPRS)	Electrochemical (CO, SO_2_, NO_2_)	Not mentioned	GPS module	HCS12/9S12 (25 MHz/12 KB/512 KB)
	In [19]	Public transportation	Wi-Fi or ZigBee or Others	DUVAS (O_3_, NO, NO_2_, SO_2_, VOCs)	Not mentioned	Not mentioned	Not mentioned
	In [99]	Car	Wi-Fi or Cellular network (GPRS)	Optical analyzer (PM), Solid-state (CO, NO_2_, NO, VOCs)	Not mentioned	GPS module	Renesas H8S (*/*/*)

**Table 10:** Summary information of the 20 systems in literature works (Part B).

Sensor Network Type	System	Operation Environment	Sensing Periodic	Number of Sensor Node in System	Geographic Coverage	Data Availability
SSN	In [105]	Outdoor roadside	200 to 300 s	60 to 200	500 m× 500 m	Email, SMS, Web App
	In [101]	Outdoor	Not mentioned	about 100	Harvard campus	Web App
	In [104]	Outdoor	Not mentioned	300 to 1200	Port Louis	Not mentioned
	In [102]	Outdoor roadside	10 min	9	Intersection circle of Keelung Road and Roosevelt Road	Researcher only
	In [108]	Indoor	5 to 60 s	Not mentioned	One floor of a building	Web page
	In [107]	Indoor	Adaptive	36	One floor of a building	None
	In [103]	Outdoor	1 min	4	1 Km${}^{2}$	Web App, mobile App
CNN	In [10]	Outdoor roadside	5 s	Not mentioned	Citywide	Web App, mobile App
	In [111]	Outdoor	Not mentioned	Not mentioned	Not mentioned	Not mentioned
	In [109]	Indoor	6 s	Not mentioned	One floor of a building	Web App, mobile App
	In [112]	Outdoor	1 s	Not mentioned	Citywide	Web page, mobile App
	In [97]	Outdoor	Not mentioned	Not mentioned	Not mentioned	Web App, mobile App
	In [113]	Outdoor	Not mentioned	Not mentioned	Not mentioned	Web App, mobile App
VSN	In [115]	Outdoor roadside	Not mentioned	1	Not mentioned	None
	In [11]	Outdoor roadside	1 min or few times per hour	Not mentioned	Citywide	Web App
	In [98]	Outdoor roadside	3 s	16	National Chiao-Tung University campus	Web App
	In [117]	Outdoor roadside	5 s	2	Citywide	Web App
	In [118]	Outdoor roadside	Not mentioned	1	American University of Sharjah campus	Web App
	In [19]	Outdoor roadside	1 min	18	Not mentioned	None
	In [99]	Outdoor roadside	Not mentioned	1	Nanyang Technological University and neighboring industrial estate	Web App

## 5. Comparison of The Three Types of Sensor Networks

In this section, the comparisons between SSN, CSN and VSN are presented. The six properties for comparison are listed as follows. Each property is described in detail with respect to the **Ranking** (the ranking of SSN, CSN and VSN of specific property, the higher the better), **Reasons** (reasons for why we choose this property for comparison) and **Explanation** (detail explanation of the ranking).

### 5.1. Mobility/Geographic-Coverage

***Ranking:*** VSN > CSN > SSN

**Reasons:**

The mobility of the carrier enables a sensor node to cover sufficient large geographic areas within a short period of time. Higher spatial resolution of the sensed data can be achieved and fewer number of sensor nodes are required compared with systems using stationary carriers.

**Explanation:**

The sensor nodes carried by the public transportations in VSN systems are with the highest mobility among the three types of sensor networks. Following is the sensor nodes carried by the public users in CSN systems because the users travel much slower than the vehicles and the users spend most of time indoor or inside cars [114]. The stationary sensor nodes in SSN systems are with the lowest or zero mobility. Intuitively, the geographic coverage of a sensor node is proportional to the mobility of the carrier.

### 5.2. Temporal Resolution

***Ranking:*** SSN > VSN > CSN

**Reasons:**

One of the objectives of TNGAPMS is to increase the temporal resolution of the acquired air pollution information. And the air pollution information from all TNGAPMSs has much higher temporal resolution than that from the conventional monitoring systems. However, the temporal resolutions of the acquired pollution information in SSN, CSN and VSN are slightly different due to several reasons.

**Explanation:**

In terms of building a pollution map, the pollution information from SSN systems has the highest temporal resolution. Then comes the pollution information from VSN systems, followed by that from CSN systems. The ranking is based on the assumptions that the sensor nodes’ sensing rates are identical in different systems and the sensors have a limited effective coverage [99]. In a single sensor node case, the pollution information’s temporal resolution of a specific location (a circular area with a specific radius) in SSN systems is the sensor node’s sensing rate itself. However, in VSN and CSN systems, the pollution information’s temporal resolutions at a specific location depend on how frequent the location is visited and how often the pollution data are sensed at that location. Intuitively, the mobility of VSN and CSN systems lowers the temporal resolutions of the acquired pollution information. Moreover, the pollution information’s temporal resolution is further reduced by redundant sampling issues like traffic jams, parked vehicles and indoor stay of users (In this case, the average temporal resolutions of SSN, CSN and VSN systems are compared. In SSN systems, only one location is monitored. In CSN and VSN systems, one sensor node typically covers several locations and this results in lower average temporal resolutions when redundant sampling issues happened).

In terms of monitoring personal exposure, the pollution information’s temporal resolution for people wearing the sensor nodes in CSN systems is the highest. For people without carrying the sensor nodes, the temporal resolution of the pollution data on personal exposure depends on the pollution map.

### 5.3. Cost Efficiency

***Ranking:*** CSN > VSN > SSN

**Reasons:**

The air pollution situation in rapid industrializing countries is much more critical than that in industrialized countries [38]. Several pollution sources (over-polluting industry, poorly tuned diesel engines and burning of trash) in developing countries contribute to the air pollution much more significantly than that in developed countries [111]. Moreover, the governments in developing countries spend less fraction of their GDPs on environmental protection than developed countries [119]. In a word, the environmental protection agencies in developing countries are dealing with serious air pollution situation with little amount of money. Hence, the cost efficiency of the air pollution monitoring system becomes a non-negligible property for comparison.

**Explanation:**

In sensor node level, the CSN systems have the highest cost efficiency, followed by the SSN systems and the VSN systems. In CSN systems, the users’ cellphones are fully utilized, including the build-in GPS and wireless communication modules, and the computational powers. The sensor nodes in CSN systems typically require no locating, communicating and computing devices and hence the cost efficiency is enhanced. In SSN systems, the stationary sensor nodes require no locating device but the communicating and computing devices because the location of a specific sensor node is known once it is deployed. For the sensor nodes in VSN systems, the GPS modules are essential due to the mobility of the carriers. Also, the communicating and computing devices are needed. Hence, the cost efficiency of VSN systems is the lowest in sensor node level.

In system level, the vast majority of the system cost is contributed by the acquisition of sensor nodes. Moreover, if the number of sensor nodes in system enlarges, a larger database for data storage and management, a faster wireless sensor network for data transmission and a more powerful computing center for data processing and decision making in real-time are required. As described in Subsection 5.1, the SSN systems require the largest amount of sensor nodes to cover a specific area, followed by the CSN systems and the VSN systems. Hence, in system level, the VSN systems have the highest cost efficiency followed by the CSN systems and the SSN systems.

The final ranking is achieved by averaging the rankings in sensor node level and system level.

### 5.4. Endurance

***Ranking:*** SSN > VSN > CSN

**Reasons:**

The endurance of the sensor nodes is a major property for comparison because it will further influence the Maintenance property and the Data Quality property. A sensor node with energy constraint (e.g., powered by a small capacity battery) requires replacing battery frequently, which increases the burden of maintenance. Moreover, the energy constraint of sensor nodes limits the use of conditioning appliances (e.g., temperature controllers, humidity controllers, gas pumps, *etc.*) that help improve the data quality.

**Explanation:**

The sensor nodes in CSN systems are with the lowest duration compared to the sensor nodes in VSN systems and SSN systems because they are powered by cellphone or portable batteries. In VSN systems, the sensor nodes are powered by vehicles’ batteries. The power supply is guaranteed once the vehicle started. In SSN systems, the sensor nodes are powered by large capacity batteries, energy harvest devices or even power lines. The duration of the sensor nodes can be counted as infinity if they are powered by power lines. Hence, the sensor nodes in SSN systems are with the highest duration compared to the sensor nodes in CSN systems and VSN systems.

### 5.5. Maintenance

***Ranking:*** VSN > SSN > CSN

**Reasons:**

In order to guarantee the data quality, maintenance on the sensor nodes like changing dead batteries, replacing malfunction components or calibrating sensors are indispensable. As a matter of fact, all TNGAPMSs require massive deployment of the sensor nodes and the sensors used in these systems need frequent calibration to be efficient [83]. We expect that, in real-life large-scale deployment, the maintenance on the sensor nodes will occupy the vast majority of efforts of the maintenance on the whole system. The feasibility of maintenance on the sensor nodes is critical in this case.

**Explanation:**

In CSN systems, the sensor nodes are carried out by the public users who are lack of special knowledge and equipment or even unlikely to explicitly maintain the sensor nodes. Moreover, it is infeasible for the professionals to collect and maintain all the sensor nodes frequently. Hence the sensor nodes in CSN systems are typically not well maintained. In SSN systems, the sensor nodes are well maintained by the professionals. However, the professionals need to visit all locations with sensor node deployment to conduct the maintenance periodically. Tremendous amount of manpower and time are required in this case and the flexibility of maintenance is reduced compared to VSN systems. In VSN systems, the sensor nodes carried by the public transportations can be driven to a specific location on demand for maintenance by the professionals. Manpower and time are saved because the professionals are able to maintain large amount of sensor nodes simultaneously. As a result, the flexibility of maintenance of VSN systems is the highest among these three types of systems.

### 5.6. Data Quality

***Ranking:*** SSN > VSN > CSN

**Reasons:**

Good data quality is essential for developing TNGAPMSs. The data quality of the low-cost portable ambient sensors used in TNGAPMSs is poorer than that of the high-end instruments used in the conventional air pollution monitoring systems. However, the low-cost portable ambient sensors still provide a fair enough accuracy and detection range [46], and flexibility in massive deployment.

**Explanation:**

In CSN systems, the constraints on weight, size and power consumption (usually powered by small capacity batteries) of the sensor nodes are extraordinary serious. These sensor nodes are typically light weight and small size, and impossible to equip with assisting instruments like temperature and humidity controllers. Moreover, the sensor nodes are not well maintained and may be put into bags or pockets that further lower the data quality. In VSN systems, these constraints are not as critical as that in CSN systems. Adding assisting tools to the sensor nodes is possible and the sensor nodes are well maintained by the professionals. However, the high mobility of the sensor nodes becomes a major factor affecting the accuracy of the sensor readings due to the varying air flow around the sensor head [10]. In SSN systems, limitations on the weight, size and power consumption (powered by power line or renewable energy source) of the sensor nodes are relaxed. The sensor nodes are able to equip with assisting equipment to ensure the data quality. The network connectivity and the sensor node’s power supply are guaranteed and the reliability of the sensed data is improved due to stationary characteristic. The data quality of SSN systems is the highest among these three types of systems, followed by the data quality of VSN systems and CSN Systems.

**Figure 11:**
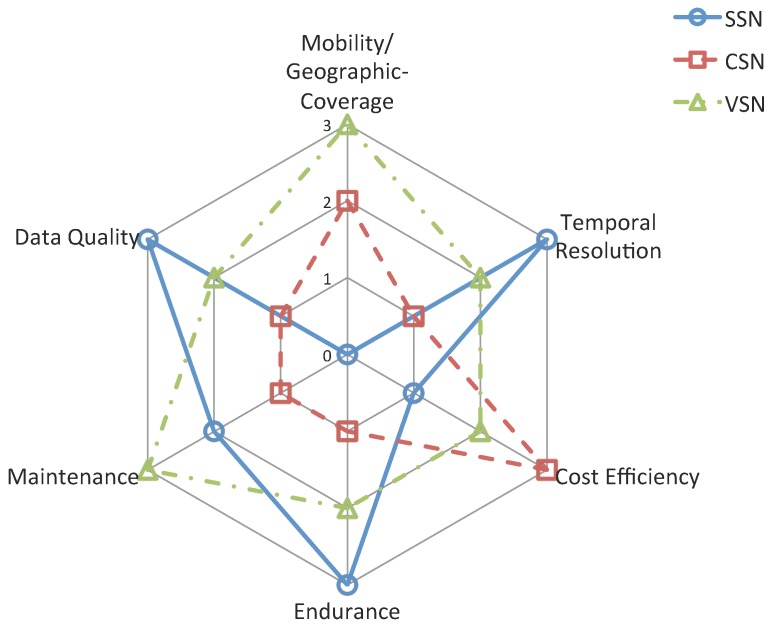
Grading result of the six major comparison properties in Static Sensor Network (SSN), Community Sensor Network (CSN) and Vehicle Sensor Network (VSN) (‘0’ means ‘None’; ‘1’ means ‘Low/Short/Inconvenient’; ‘2’ means ‘Medium’ and ‘3’ means ‘High/Long/Convenient’).

After we have an in-deep understanding of these six comparison properties described above, we graded these properties of SSN, CSN and VSN systems using the grading code defined as: `0’ means ‘None’; ‘1’ means `Low/Short/Inconvenient’; ‘2’ means ‘Medium’ and ‘3’ means ‘High/Long/Convenient’. The final grade of each comparison property of SSN, CSN and VSN systems following the descriptions above are shown in Figure 11.

## 6. Discussion and Conclusions

Air pollution is an essential environmental issue due to the tremendous impacts on public health, global environment, and worldwide economy. Urban air pollution with non-uniform distribution trend arises the necessity for pollution monitoring with high spatio-temporal resolution, which the conventional air pollution monitoring systems cannot provide because of the limited data availability and non-scalability of the systems. By utilizing the advance sensing technologies, MicroElectroMechanical Systems (MEMS) and Wireless Sensor Network (WSN), researchers are pushing the concept of The Next Generation Air Pollution Monitoring System (TNGAPMS) to the limit and have achieved great progresses. Many of state-of-the-art air pollution monitoring systems have been implemented and tested. All of these systems evidence that an air pollution monitoring system with high spatio-temporal resolution, cost and energy efficiency, deployment and maintenance feasibility, convenient accessing ability for the public or professional users are achievable. However, from Section 4 and Section 5, we can conclude that there are still some issues or challenges of these existing systems that need to be addressed. Also there are some abilities or characteristics of these existing systems that we want to carry forward or enhance when building the future systems.

### 6.1. Issues and Challenges Need to Be Addressed

***Lack of 3-Dimensional Data Acquisition Ability:*** All the systems presented in Section 4 are only able to monitor the air pollution situation of urban surface or roadside while the necessities and importance of the 3-Dimensional air pollution information are highlighted [120,121,122]. Current LIDARs or satellites based 3-Dimensional monitoring systems face the same issues as the conventional monitoring systems. We anticipate that 3-Dimensional air pollution information with high spatio-temporal resolution can be acquired in real-time by mounting the portable sensor nodes on the multi-rotors Unmanned Aerial Vehicles (UAVs).

***Infeasibility of Active Monitoring:*** The sensor nodes in SSN, CSN and VSN systems presented are all passive monitoring sensor nodes (sensor nodes periodically update data). We believe that active monitoring (users can fully control the sensor network including the formation and routes of the sensor nodes) provides higher flexibility and QoS.

***Uncontrolled or Semi-Controlled Carriers:*** The carriers in SSN, CSN and VSN systems are uncontrolled or semi-controlled because they are either stationary or with pre-determined routes. We anticipate that fully controlled carriers have higher mobility and make active monitoring possible. By utilizing the fully controlled carriers (*i.e.*, the multi-rotors UAVs), feasibility in deployment, cost efficiency of systems and convenience in maintenance can be achieved.

### 6.2. Abilities and Characteristics Need to Be Carried Forward

In fact, all the abilities or characteristics of these state-of-the-art TNGAPMSs need to be carried forward and some of them can be improved in future systems.

***Mobility of Carriers:*** The mobility of the carriers enables one sensor node to cover a sufficient large geographic area within a short period of time. The number of sensor nodes required is reduced and the system cost and maintenance are relaxed. In fact, the multi-rotors (There are regulations for drones in some areas and we put aside this issue for a moment in this paper.) will not be trapped into traffic-jams or stop by unreachable areas as the carriers in VSN systems. The multi-rotors have much higher mobility than the carriers in VSN systems.

***Feasibility of Maintenance:*** The system’s feasibility on maintenance need to be carried forward and enhanced because it affects the data quality and cost efficiency of the system. If the fully controlled UAVs were utilized in the system, professional maintenance on large number of sensor nodes can be performed simultaneously by driving all UAVs to a specific location. In this case, the quality of the sensed data is guaranteed while the time and manpower for maintenance are saved.

***Add-on Sensor Ability:*** We note that all the sensor nodes in the existing TNGAPMSs are with no add-on ability. Reconfigurations on the hardware and software of the sensor nodes are needed whenever the sensing species are modified. In real-life large-scale applications, there could be hundreds or even thousands of sensor nodes in the system. Sensor nodes with add-on (the sensor node is able to identify the type of sensor mounted and chooses the suitable program to handle the sensing data) ability are essential in this case. Properties like modifiable sensing and transmitting intervals, remote programmable ability, cost and energy efficiencies and failure check feature are also essential.

Last but not least, all the existing state-of-the-art TNGAPMSs claim that they have a better spatio-temporal resolution than the conventional air pollution monitoring systems (which is obvious). However, none of them has ever considered how good they are, not to mention the comparisons among the SSN, CSN and VSN systems, with respect to real-time performance, spatio-temporal resolution and QoS. And this will be a major direction of our future works.
